# Supplementary material for: The RNA-binding protein vigilin regulates VLDL secretion through modulation of Apob mRNA translation
Source: Nat Commun. 2016 Sep 26;7:12848. doi: 10.1038/ncomms12848 (PMC5052685; doi:10.1038/ncomms12848)
Supplement: Supplementary Information — Supplementary Figure 1-8 and Supplementary Table 1-2 [file ncomms12848-s1.pdf]

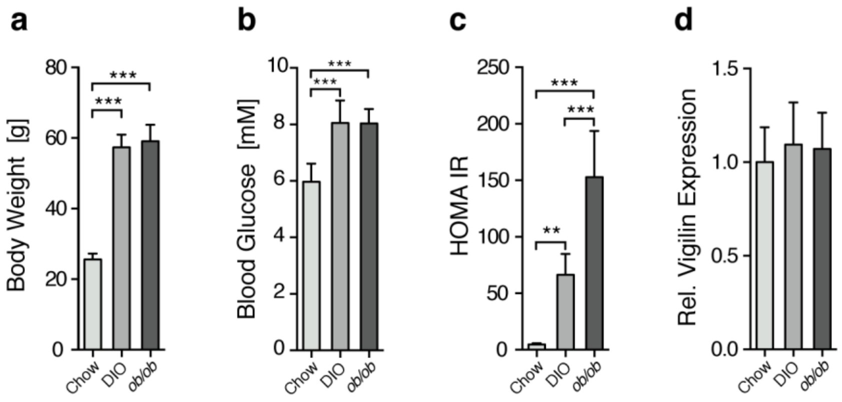

Supplementary Figure 1: Additional metabolic parameters of obesity mouse models and controls. (**a**) Body weight, (**b**) blood glucose and (**c**) insulin resistance index of homeostatic model assessment (HOMA IR) of 20 week-old chow fed (Chow), diet-induced obese (DIO) C57BL/6 mice, and 23 week old *ob/ob* mice under study (n = 6 per group). (**d**) qPCR analysis of hepatic vigilin transcript levels. Data are shown as relative expression levels. All values are expressed as mean ± s.d. $^{**}P \le 0.01$, $^{***}P \le 0.001$; *p*-values were determined ANOVA with Holm-Sidak *post hoc* analysis.

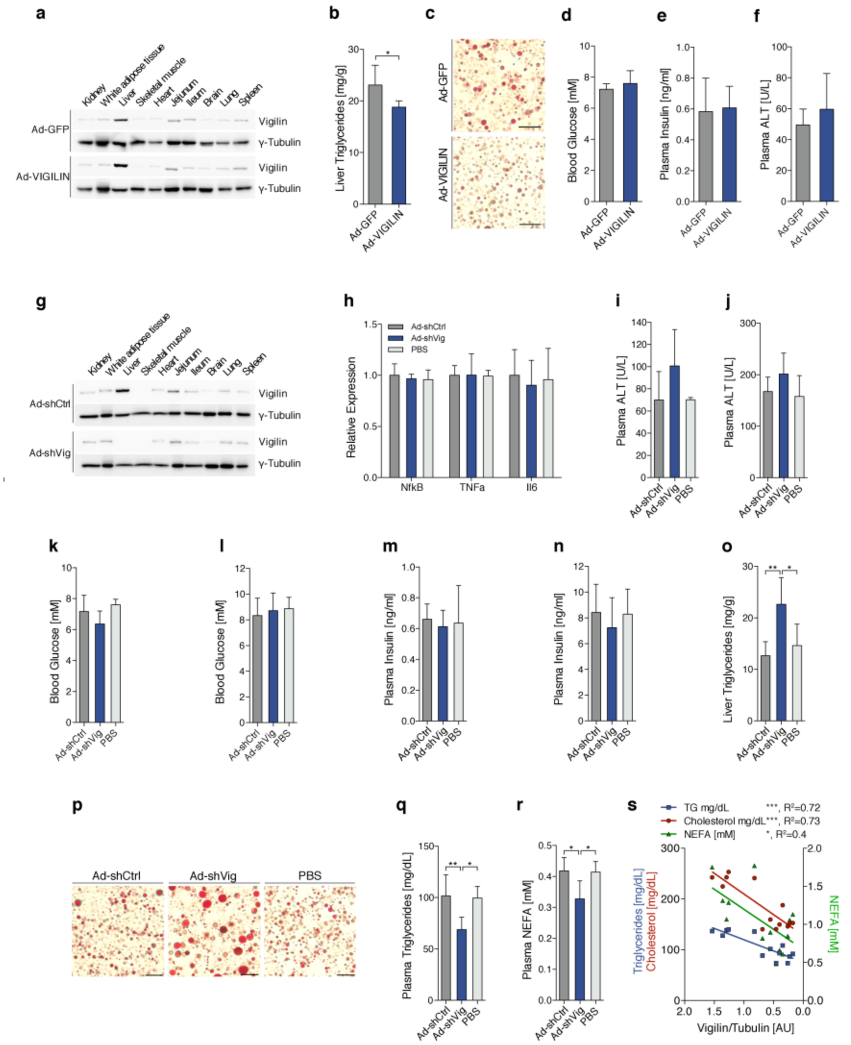

Supplementary Figure 2: Gene expression and metabolic parameters following overexpression or silencing of vigilin. (**a-f**) Male C57BL/6 mice, 8 weeks of age, were injected with either Ad-GFP (n = 5) or Ad-VIGILIN (n = 5) and studied 7 days postinjection. (**a**) Multiple tissue immunoblot, (**b**) liver triglycerides, (**c**) liver oil red O stainings (scale bar = 10µm), (**d**) blood glucose, (**e**) insulin and (**f**) alanine transaminase (ALT) levels. (**g-r**) 10 week old chow fed and 20 week old diet-induced obese C57BL/6 mice (DIO) were injected with either Ad-shCtrl (n = 6, chow diet; n = 8, DIO), Ad-shVig (n = 6, chow diet; n = 8, DIO) or PBS (n = 3, chow diet and DIO) and studied 10 days postinjection. (**g**) Multiple tissue immunoblot indicating, (**h**) qPCR analysis of liver inflammation markers *NfkB*,

TNFa and *IL6* and plasma ALT levels in (**i**) chow fed and (**j**) DIO mice. Blood glucose in (**k**) chow fed and (**l**) DIO mice as well as plasma insulin levels in (**m**) chow fed and (**n**) DIO mice. (**o**) Liver triglyceride levels and (**p**) respective liver oil red O stainings of chow fed mice (scale bar = 10µm). (**q**) Blood triglyceride and (**r**) non-esterified fatty acid (NEFA) levels in chow fed mice injected with Ad-shVig and control Ad-shCtrl or PBS. (**s**) Reversed correlation of hepatic vigilin levels with plasma triglycerides, NEFA and cholesterol levels upon adenovirus-induced knockdown in DIO mice. All values are expressed as mean ± s.d. **P* ≤ 0.05, ***P* ≤ 0.01, ****P* ≤ 0.001; *P*-values were determined using student's *t*-test (b, d-f) or ANOVA with Holm-Sidak *post hoc* analysis (h-o, q, r). R^2 were determined by two-tailed Pearson's correlation test in s.

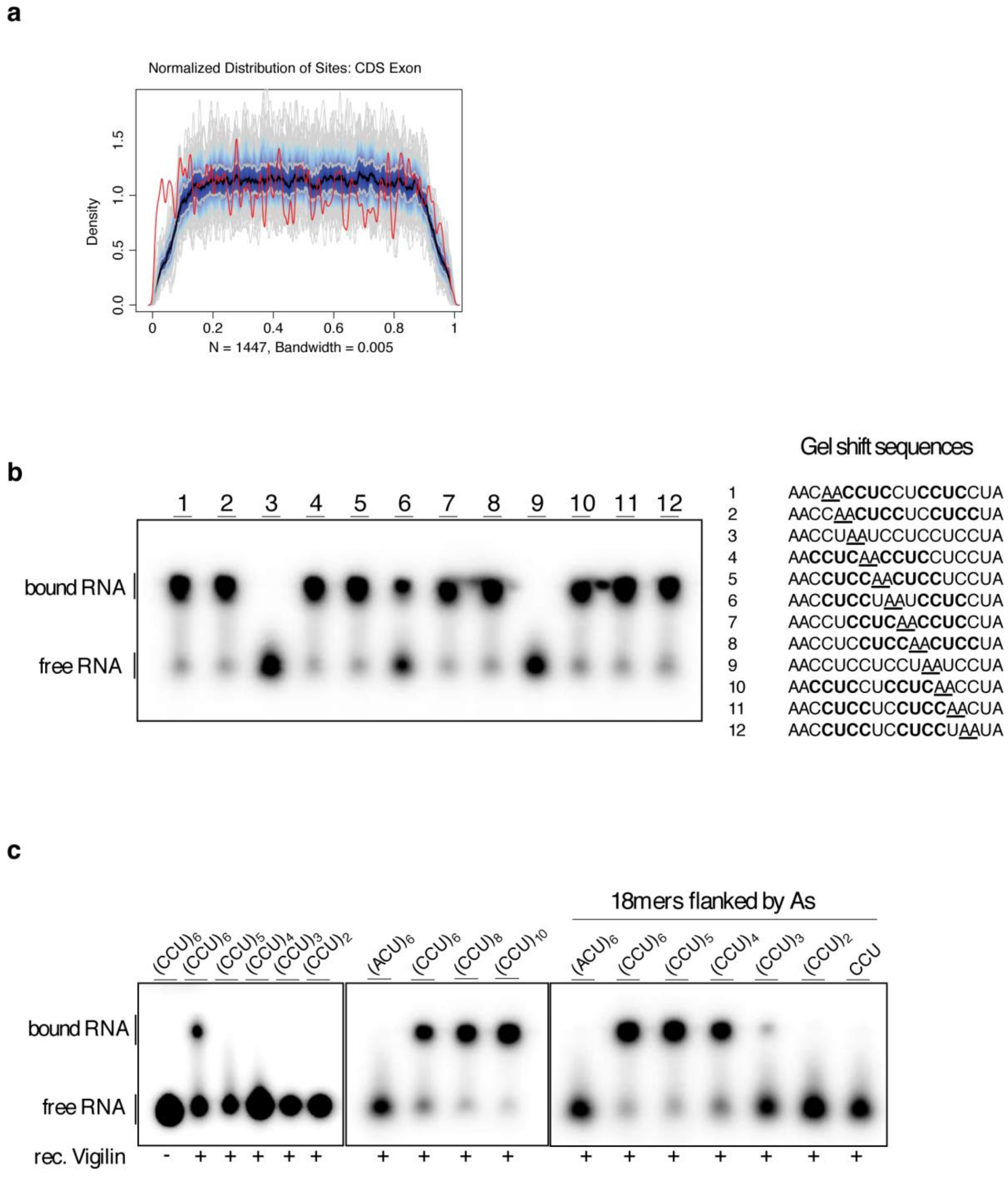

Supplementary Figure 3: Electrophoretic Mobility Shift Assays (EMSAs) using recombinant VIGILIN. (**a**) Spatial kernel density estimation of binding sites across CDS exons. On the *y*-axis the density distribution is shown, on the *x*-axis N is the number of binding sites in CDS exons (N = 1447) is shown with a smoothening

bandwidth of 5. The signal of the clusters (red line) shows an even distribution across the CDS exons within the probability range of the background distal distribution (blue and grey, color shaded according to probability percentiles). The black line indicates the average background probability distribution, dark blue indicates 50th percentile smoothened background distribution, bright blue indicates 75th percentile smoothened background distribution, grey lines indicate sampled individual background probabilities. (**b-c**) Synthetic RNAs (10 nM) were radiolabeled, incubated with 2 μM His_6-tagged VIGILIN recombinant protein and separated on a 1% agarose gel. (**b**) An 18-nt long RNA sequence containing 5x CCU-repeats was systematically mutated at two adjacent sites with As (underlined) and used for EMSAs. Shifts were only observed with sequences containing a CYYC-2nt-CYYC RRE (in which Y=C/U). CYYC-2nt-CYYC RRE is indicated in bold letters. (**c**) EMSA using CCU-trinucleotides with various repeats (left and middle panel), and repeats with A-flanking sites indicate that sequences ≥18 nt containing ≥4 CCU repeats are required for efficient RNA binding.

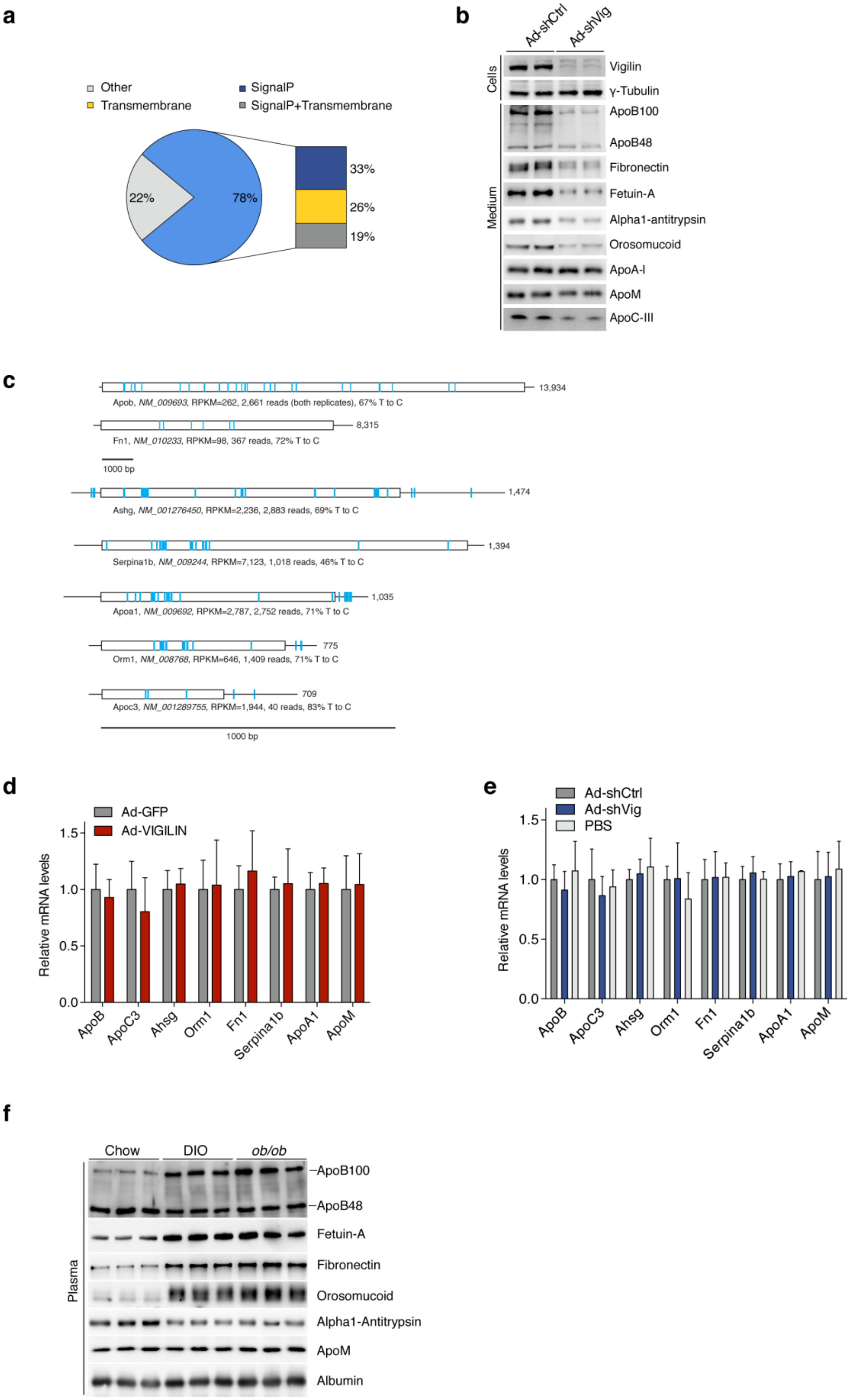

a
Other
Transmembrane
SignalP
SignalP+Transmembrane
22%
78%
33%
26%
19%
b
Ad-shCtrl
Ad-shVig
Cells
Medium
Vigilin
γ-Tubulin
ApoB100
ApoB48
Fibronectin
Fetuin-A
Alpha1-antitrypsin
Orosomucoid
ApoA-I
ApoM
ApoC-III
c
13,934
Apob, NM_009693, RPKM=262, 2,661 reads (both replicates), 67% T to C
8,315
Fn1, NM_010233, RPKM=98, 367 reads, 72% T to C
1000 bp
1,474
Ashg, NM_001276450, RPKM=2,236, 2,883 reads, 69% T to C
1,394
Serpina1b, NM_009244, RPKM=7,123, 1,018 reads, 46% T to C
1,035
Apoa1, NM_009692, RPKM=2,787, 2,752 reads, 71% T to C
775
Orm1, NM_008768, RPKM=646, 1,409 reads, 71% T to C
709
Apoc3, NM_001289755, RPKM=1,944, 40 reads, 83% T to C
1000 bp
d
Ad-GFP
Ad-VIGILIN
Relative mRNA levels
1.5
1.0
0.5
0.0
ApoB
ApoC3
Ahsg
Orm1
Fn1
Serpina1b
ApoA1
ApoM
e
Ad-shCtrl
Ad-shVig
PBS
Relative mRNA levels
1.5
1.0
0.5
0.0
ApoB
ApoC3
Ahsg
Orm1
Fn1
Serpina1b
ApoA1
ApoM
f
Chow
DIO
ob/ob
Plasma
ApoB100
ApoB48
Fetuin-A
Fibronectin
Orosomucoid
Alpha1-Antitrypsin
ApoM
Albumin

Supplementary Figure 4: Characterization and regulation of strongest vigilin targets. (**a**) 78 of the top 100 vigilin targets identified by PAR-CLIP are secretory pathway proteins either containing a signal peptide (SignalP; 33), ≥1 transmembrane domains (26) or both (19). (**b**) Validation of MS-LFQ data by immunoblotting of six targets from medium of primary hepatocytes isolated from mice that were injected with either Ad-shCtrl or Ad-shVig. ApoM and apoA-I were used as loading controls. (**c**) Schematic representation illustrating distribution of vigilin binding sites on mRNAs under study as identified by PAR-CLIP. Blue lines indicate sites of T-to-C conversion. Gene name, identifier, RPKM and total number of reads as well as T-to-C conversion rates are indicated. (**d, e**) qPCR analysis of secreted mRNA targets among top 100 with highest downregulation of protein levels upon (**d**) overexpression (n = 6 per group) and (**e**) knockdown of vigilin (n = 6, Ad-shCtrl; n = 6, Ad-shVig; n = 3, PBS). Data are shown as relative expression levels and as the mean ± s.d. Statistical significance was assessed by student's *t*-testing (in **d**) or ANOVA with Holm-Sidak *post hoc* analysis (in **e**). All data are shown as the mean ± s.d. (**f**) Immunoblot analysis of targets from blood plasma in 20 week-old chow fed (Chow) or high-fat diet C57BL/6 (DIO) and in 23 week old *ob/ob* mice.

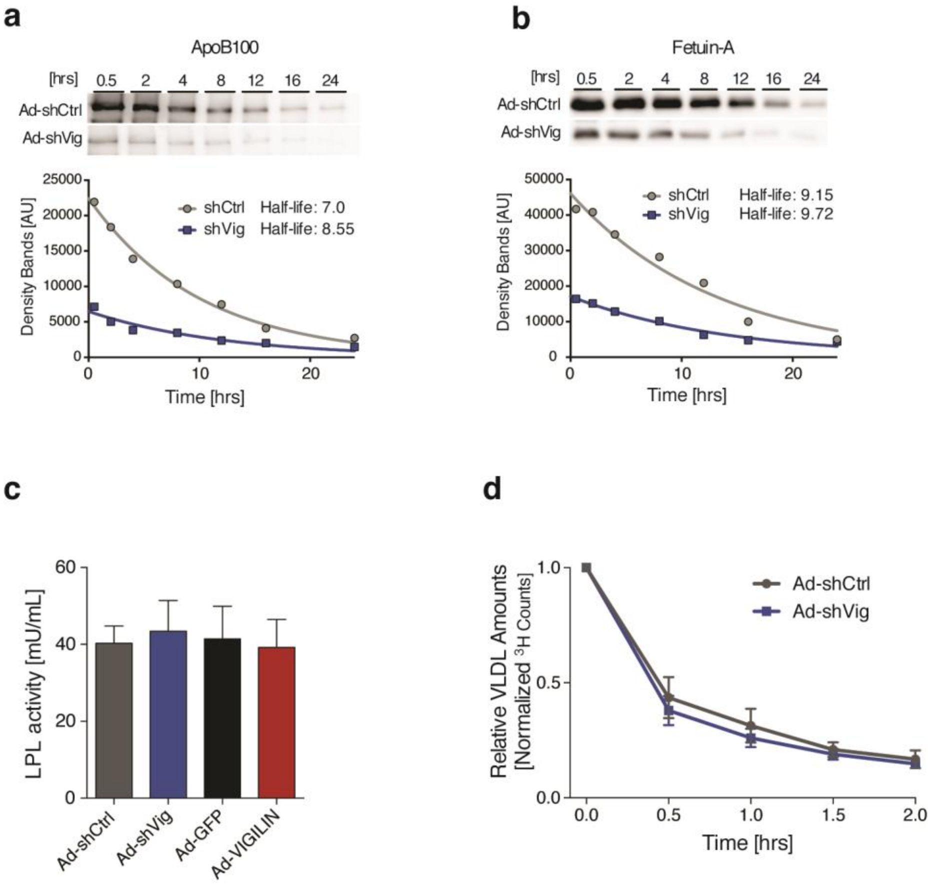

Supplementary Figure 5: Characterization of apoB, fetuin-A and VLDL and triglyceride clearance. (**a, b**) Half-lives of apoB and fetuin-A remained unchanged upon vigilin knockdown. Primary hepatocytes were treated with cycloheximide for translational inhibition and harvested at indicated time points for half-life assessment of (**a**) apoB and (**b**) fetuin-A by immunoblotting (upper panel). Lower panel: Graphs illustrate densitometric readout from band intensities. (**c**) LPL-activities from overexpression and knockdown experiments (n = 6 per group). (**d**) VLDL clearance upon hepatic knockdown of vigilin using 10 week old mice. ^{3}H-radiolabeled VLDL was injected into mice treated with Ad-shCtrl or Ad-shVig (n = 6 per group) prior to blood collection at indicated time points postinjection. Extracted blood plasma samples were subjected to scintillation-counting for quantification of VLDL clearance rates. Statistical significance was assessed by student's *t*-testing or ANOVA with Tukey's *post hoc* analysis. Data in **c** and **d** are shown as the mean ± s.d.

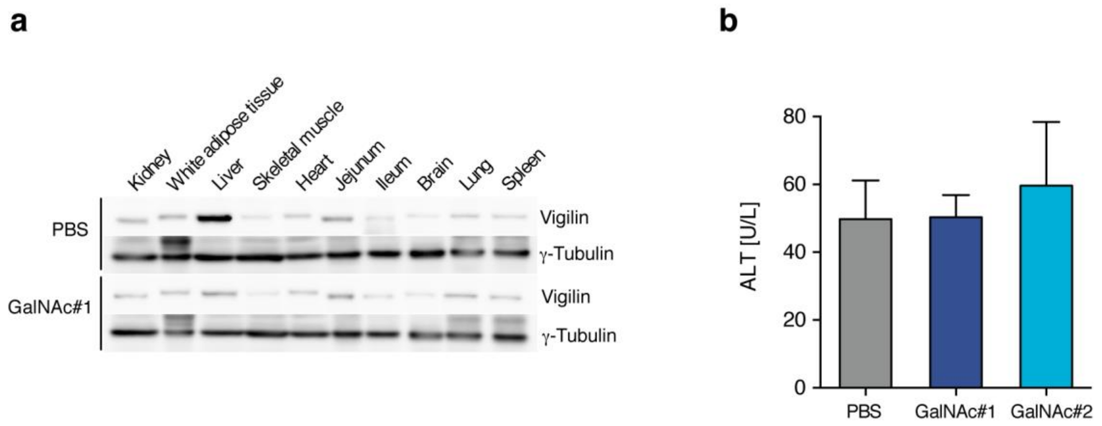

Supplementary Figure 6: Characterization of long-term knockdown of hepatic vigilin in *Ldlr*$^{-/-}$ mice. (**a**) Tissue panel indicating liver specific knockdown of vigilin from mice treated with siVig-GalNAc#1 as opposed to PBS treated mice for 18 weeks starting at 4 weeks of age.. (**b**) Blood alanine transaminase (ALT) levels after 18 weeks of treatment with PBS, GalNAc#1 or GalNAc#2 (n = 8 per group). Statistical significance was determined by ANOVA with Holm-Sidak *post hoc* analysis. All data are shown as the mean ± s.d.

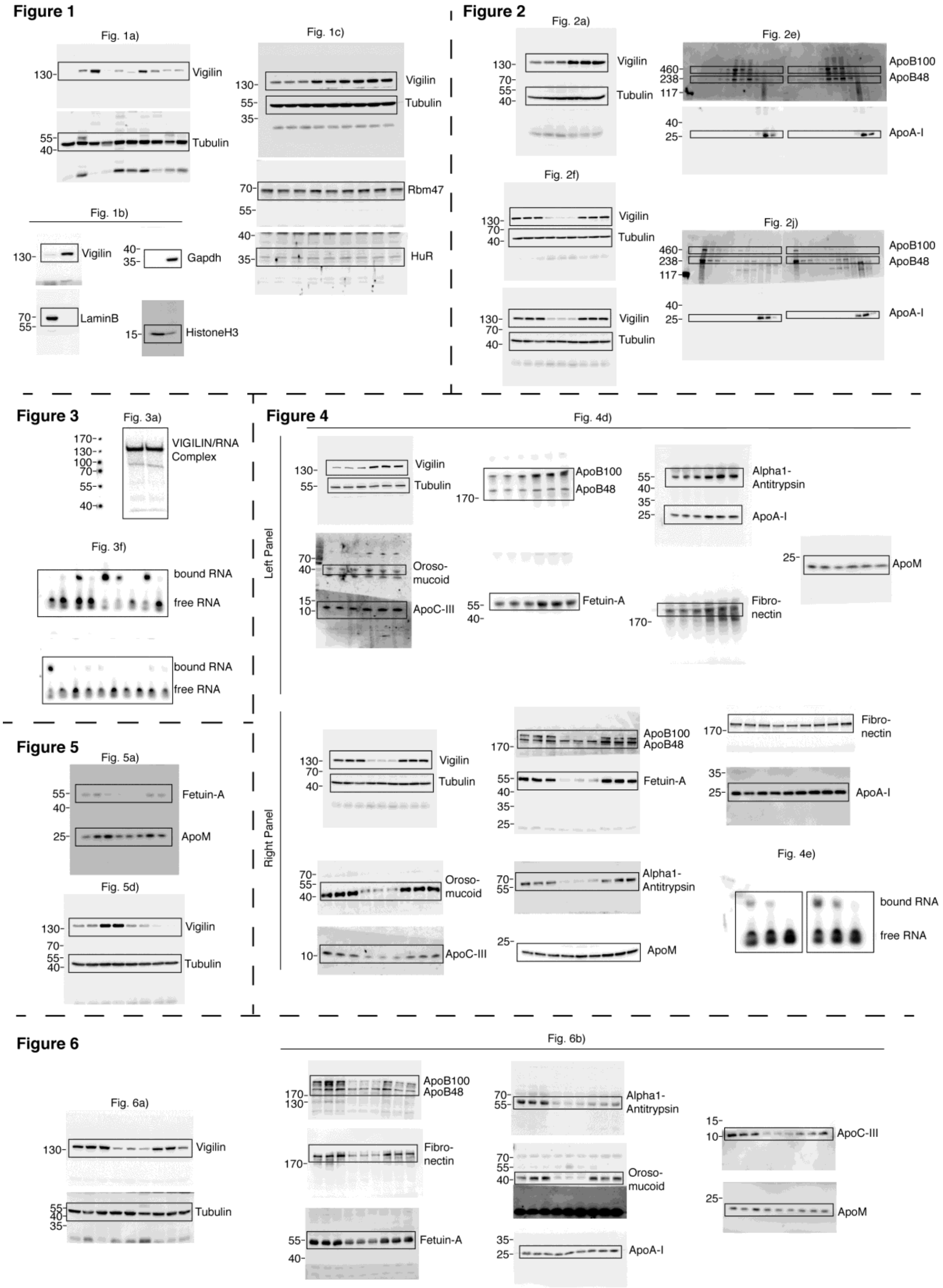
Figure 1
Fig. 1a)
Vigilin
Tubulin
Fig. 1b)
Vigilin
Gapdh
LaminB
HistoneH3
Fig. 1c)
Vigilin
Tubulin
Rbm47
HuR
Figure 2
Fig. 2a)
Vigilin
Tubulin
Fig. 2e)
ApoB100
ApoB48
ApoA-I
Fig. 2f)
Vigilin
Tubulin
Fig. 2j)
ApoB100
ApoB48
ApoA-I
Vigilin
Tubulin
Figure 3
Fig. 3a)
VIGILIN/RNA Complex
Fig. 3f)
bound RNA
free RNA
bound RNA
free RNA
Figure 4
Fig. 4d)
Left Panel
Vigilin
Tubulin
ApoB100
ApoB48
Alpha1-Antitrypsin
ApoA-I
ApoM
Oroso-mucoid
ApoC-III
Fetuin-A
Fibro-nectin
Right Panel
Vigilin
Tubulin
ApoB100
ApoB48
Fetuin-A
Fibro-nectin
ApoA-I
Oroso-mucoid
Alpha1-Antitrypsin
ApoC-III
ApoM
Fig. 4e)
bound RNA
free RNA
Figure 5
Fig. 5a)
Fetuin-A
ApoM
Fig. 5d)
Vigilin
Tubulin
Figure 6
Fig. 6a)
Vigilin
Tubulin
Fig. 6b)
ApoB100
ApoB48
Fibro-nectin
Fetuin-A
Alpha1-Antitrypsin
Oroso-mucoid
ApoA-I
ApoC-III
ApoM

Supplementary Figure 7: Uncropped images of blots used in main figures. The number of the corresponding figure is shown above the panels. Regions used for figures are boxed. The position of the molecular mass markers is shown on the left in kDa.

Suppl. Fig. 2a)

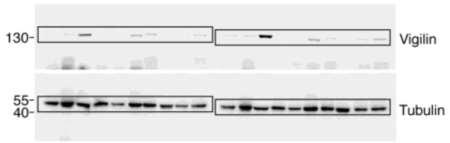

Suppl. Fig. 2g)

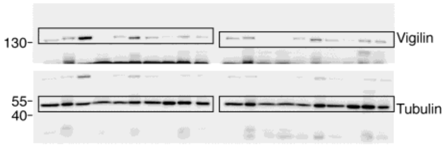

Suppl. Figure 3

Suppl. Fig. 3b)

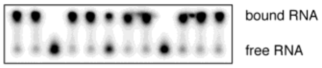

Suppl. Fig. 3c)

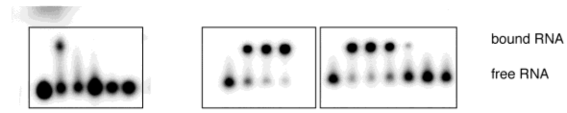

Suppl. Figure 4

Suppl. Fig. 4b)

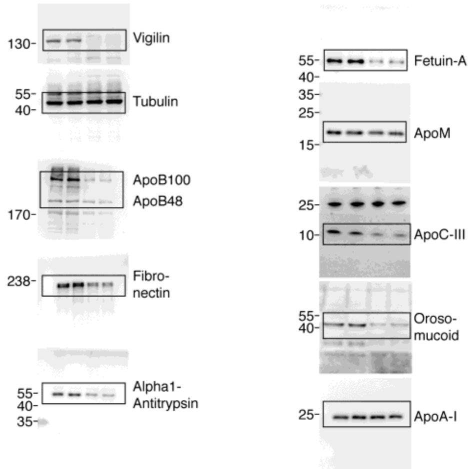

Suppl. Fig. 4f)

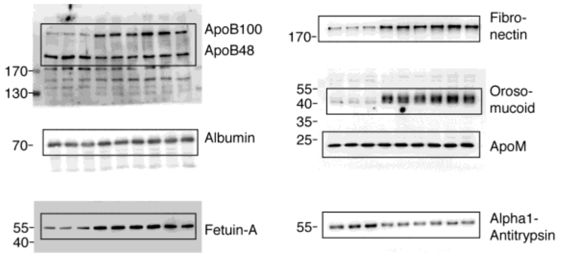

Suppl. Figure 5

Suppl. Fig. 5a)

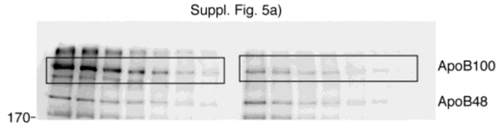

Suppl. Fig. 5b)

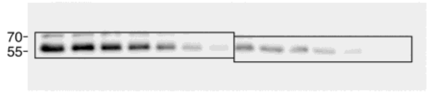

Suppl. Figure 6

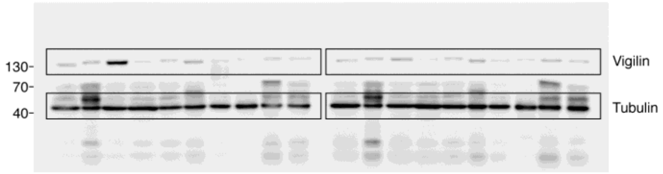

Supplementary Figure 8: Uncropped images of blots and gels used in supplemental figures. Number of the corresponding figure is shown above the panels. Regions used for figures are boxed. Position of the molecular mass markers is shown on the left in kDa.

Diagnosis	n	%Steatosis	BMI	AST (U/L)	ALT (U/L)
Healthy	5	2.0 ± 2.7	23.7 ± 2.6	34.2 ± 19.1	34.0 ± 18.7
NAFLD	10	38.5 ± 21.9	32.3 ± 5.5	18.2 ± 21.5	17.9 ± 23.1
NASH	10	59.0 ± 2.7	33.0 ± 3.3	43.8 ± 12.0	67.8 ± 35.6

Supplementary Table 1: Human data including patients with non-alcoholic fatty liver disease (NAFLD) and non-alcoholic steatohepatitis (NASH). Liver biopsies from patients under study for VIGILIN expression (main figure) and their corresponding body mass index (BMI), degree of steatosis as well as blood alanine (ALT) and aspartate (AST) transaminase levels. All values are shown as mean ± s.d.

Supplementary Table 2

Oligo Sequences used for qPCR

Gene	Fw/Rev	Sequence
Serpina1b	Forward	CAATGGGGCTGACCTCTCT
	Reverse	GCACAGCCTTATGCACAGC
Fn1	Forward	CGGAGAGAGTGCCCCTACTA
	Reverse	CGATATTGGTGAATCGCAGA
ApoA1	Forward	TATGTGGATGCGGTCAAAGA
	Reverse	TGAACCCAGAGTGTCCCAGT
Orm1	Forward	ATTTGTCGACTGGAAAAAGGAT
	Reverse	TGAGTTGGGGAAGGAGACC
ApoM	Forward	CCCAGACATGAAAACAGACCT
	Reverse	GGGTGTGGTGACCGATTG
Hdlbp	Forward	TCTGGCTAAAGATCAAGGTCTCT
	Reverse	GCCAATAACAAAGCGATGGTG
Ahsg	Forward	ATACCAACGTGGTCCACACC
	Reverse	AACTCCACCAGAGTAGACACTGG
Apob	Forward	TGTACAACTGGTCAGCCTCCTACAC
	Reverse	TGGTGTAGAGATCCATCACAGGAC
Actb	Forward	CTAAGGCCAACCGTGAAAAG
	Reverse	ACCAGAGGCATACAGGGACA
Apoc3	Forward	CGCTAAGTAGCGTGCAGGA
	Reverse	GGGATCTGAAGTGATTGTCCA
Actb	Forward	CTAAGGCCAACCGTGAAAAG
	Reverse	ACCAGAGGCATACAGGGACA
Nfkb	Forward	GACCACTGCTCAGGTCCACT
	Reverse	TGTCACTATCCCGGAGTTCA
Tnfa	Forward	AAATGGCCTCCCTCTCATCA
	Reverse	AGATAGCAAATCGGCTGACG
Il6	Forward	TGATGGATGCTACCAAACTGG
	Reverse	TTCATGTACTCCAGGTAGCTATGG

GalNAc conjugated siRNAs

GalNAc#1	Sense:	5'-3' GfsasGfaUfcAfaCfAfUfuGfaCfcAfuAfaAfL96
	Antisense:	5'-3' usUfsuAfuGfgUfcAfaugUfuGfaUfcUfcsusa
GalNAc#2	Sense:	5'-3' AfsgsGfaAfgAfuCfGfGfgCfuUfuAfaGfgAfL96
	Antisense:	5'-3' usCfscUfuAfaAfgCfccgAfuCfuUfcCfusgsc
	Nf	= 2' Fluoro bases
	n	= 2'O Methyl bases
	s	= Phosphorothioate
	L96	= GalNAc trivalent

shRNA sequences in adenovirus

sh.Ctrl	CAACAAGATGAAGAGCACCAACTCGAGTTGGTGCTCTTCATCTTGTTG
shVig#1	GCTCGCATTAAGAAGATTTATCTCGAGATAAATCTTCTTAATGCGAGC
shVig#2	CCAGAGGTTATCATCAACTTTCTCGAGAAAGTTGATGATAACCTCTGG

Supplementary Table 2: List of sequences for DNA oligos, shRNAs and GalNAc-conjugated siRNAs used in this study.
